# Resolution of Colchicine-Resistant, Corticosteroid-Dependent Acute Idiopathic Recurrent Pericarditis With Rilonacept: A Case Report

**DOI:** 10.7759/cureus.82325

**Published:** 2025-04-15

**Authors:** Satyatejas G Reddy, Samson L Mumber, Rahul Garg

**Affiliations:** 1 Medicine, Medical College of Georgia, Augusta University, Augusta, USA; 2 Medicine, Carolinas Campus, Edward Via College of Osteopathic Medicine, Spartanburg, USA; 3 Medicine, Cardiology Rome, Harbin Clinic, Rome, USA

**Keywords:** acute recurrent pericarditis, calcification of the pericardium, colchicine, rilonacept, treatment-resistant pericarditis

## Abstract

Pericarditis, the most common disease of the pericardium, is characterized by pleuritic, sharp, stabbing chest pain that worsens with breathing. Pericarditis can arise from various causes, including viral infections, malignancies, and drug reactions, though the cause often remains idiopathic. Treatment typically involves nonsteroidal anti-inflammatory drugs (NSAIDs), colchicine, and/or corticosteroids. In rare cases, biologic therapy may be required. Rilonacept, a recently approved interleukin-1 (IL-1) inhibitor for recurrent pericarditis, has shown promise in alleviating symptoms and preventing recurrence. Unlike NSAIDs, which inhibit cyclooxygenase enzymes, and colchicine, which disrupts microtubule assembly and inflammatory chemotaxis, rilonacept binds IL-1 and blocks proinflammatory signaling cascades. Additionally, while long-term corticosteroids do inhibit proinflammatory cytokines, they are known to have a host of long-term side effects, including osteoporosis and hyperglycemia. The efficacy of rilonacept across various stages of pericardial inflammation and in all recurrent cases remains uncertain. We report a case of idiopathic acute recurrent pericarditis in a 55-year-old South Asian woman. Eight months after the initial diagnosis, she experienced rising inflammatory markers and intermittent fevers despite treatment with ibuprofen and colchicine. Her condition progressed to corticosteroid dependence and marginal pericardial calcification, identified via an echocardiogram eight days after recurrent symptoms began. Symptom resolution and inflammation control were achieved with rilonacept, showing sustained success at a 12-month follow-up.

## Introduction

Pericarditis, the inflammation of the pericardium, is the most common form of pericardial disease [1]. Autoimmune conditions, viruses, and many other stimuli have been linked to acute and recurrent pericarditis, but the etiology of the disease is not well understood. In developed countries, viruses are the most common cause of pericarditis, while tuberculosis (TB) infection is the most common cause in developing countries [1]. However, more than 80% of the time, the cause of pericarditis is deemed idiopathic after conventional diagnostic approaches [2]. Idiopathic pericarditis is often assumed to be viral in origin. Because the viral mechanisms that lead to inflammation and pericarditis are poorly understood, the cause of pericarditis cannot be ascribed to a specific etiology, leading to what most clinicians deem as *idiopathic* or *presumed viral *pericarditis [3]. A common side effect of acute pericarditis is recurrent pericarditis, which is when an episode of pericarditis reoccurs at least four to six weeks after the all the symptoms from the original episode of pericarditis subside, and this occurs in 15% to 30% of people who have been diagnosed with pericarditis [4-6]. Nonsteroidal anti-inflammatory drugs (NSAIDs) and colchicine remain the first line of defense against pericarditis, with colchicine specifically being shown to reduce symptoms and recurrence in clinical trials [4,5,7,8]. If the inflammation does not respond to either medication, corticosteroids are used [1]. However, while steroids may reduce the inflammation of the pericardium momentarily, they are associated with recurrence, contribute to a longer disease progression, and must be slowly tapered off [4,9]. Additionally, long-term corticosteroid usage leads to a poor quality of life and further side effects, including osteoporosis, adrenal suppression, infection, and Cushing's syndrome [10]. If pericarditis does not respond, or if the patient is dependent on corticosteroids, interleukin-1 (IL-1) blockers such as anakinra or the relatively newer medication rilonacept can be used as a possible treatment. The innate immune system mediates inflammation through cytokines, predominantly IL-1. Patients with autoinflammatory diseases, such as recurrent pericarditis, are predisposed to having an inappropriate innate immune response. By using a medication that blocks the activity of a major cytokine within the pathway, IL-1, patients with these types of diseases are more likely to experience symptomatic relief [2]. Rilonacept was recently approved by the Food and Drug Administration (FDA) in 2021 to treat acute recurrent pericarditis due to its success in clinical trials in resolving recurrent pericarditis [11,12], but due to the small number of patients using the drug, the relative novelty, and the rarity of the disease, many questions remain about how rilonacept affects patient populations with ranging severities of pericarditis symptoms and medical histories [13-15]. Conducting large-scale studies on acute recurrent pericarditis is challenging due to its relatively low incidence and the heterogeneous nature of its clinical presentations. These factors complicate patient recruitment and the standardization of study protocols [16]. We present a case of NSAID- and colchicine-resistant acute recurrent pericarditis that remained corticosteroid-dependent. Inflammation and severity of symptoms were only resolved using the IL-1 inhibitor, rilonacept.

## Case presentation

The patient was a 55-year-old South Asian woman with a medical history of type 2 diabetes, hypertension, and hypothyroidism, all well-controlled with medication. She had no family history of cardiac disease. She had one episode of acute renal failure at 52 years old, without any history or diagnosis of chronic kidney disease, but no cause was ever determined.

**Figure 1:**
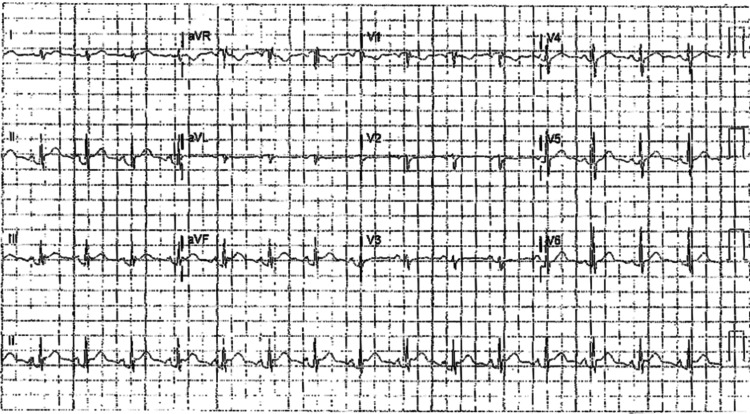
Electrocardiogram at presentation in February 2023, showing ST segment elevation followed by a PR depression.

The initial onset of symptoms began with the patient experiencing fatigue during an episode of physical exercise. Further testing the following day revealed a normal electrocardiogram (EKG) and an elevated erythrocyte sedimentation rate (ESR) of 54 mm/hour, a C-reactive protein (CRP) level of 40.4 mg/dL, a white blood cell count (WBC) of 14.2 x 10^9^/L, and neutrophil count of 11.6 K/µL. Over the next two days, constant, sharp, pleuritic chest pain developed, and the pain began to radiate to each shoulder. The patient could not tolerate any form of physical exercise. Two days following the previous labs, the patient had new labs that revealed an elevated D-dimer and a new CRP of 168.5 mg/dL, prompting the patient to be referred to the emergency room (ER). In the ER, the patient confirmed to the physician that she was still having pleuritic, anterior chest pain, which was relieved significantly upon leaning forward. Upon cardiac auscultation, the patient was also shown to have a slight pericardial rub. The EKG revealed ST segment elevation followed by a PR depression (Figure 1). The patient had a negative computed tomography angiography (CTA) for pulmonary embolism, and her cardiac enzymes were normal. Echocardiography did not show any significant abnormalities and showed normal wall motion, which ruled out myocardial infarction. No pericardial effusions were noted. Further labs showed an elevated CRP of 18.9 mg/dL, which, in conjunction with the pericardial rub and EKG results, led to the diagnosis of acute pericarditis.

A cardiovascular physician was consulted, and the patient was started on aspirin 325 mg and colchicine 0.6 mg, which relieved her acute chest pain. Over the next few days, the patient continued the colchicine 0.6 mg twice daily and was prescribed ibuprofen 800 mg three times daily, and the exertional chest pain subsided. The patient still reported dyspnea and tachycardia on exertion but stated that the severity was decreasing over time. Repeated labs showed a slightly elevated ESR but a normal CRP and WBC, indicating that inflammation had been reduced significantly. The patient continued to take colchicine 0.6 mg until June and consequently tapered it off over four weeks. She had also stopped the ibuprofen 800 mg three times daily until May due to her concern regarding her previous renal failure. Her symptoms had completely resolved. 

In October 2023, the patient began experiencing another episode of pleuritic anterior chest pain that she described as worse than the previous episode. The patient experienced fatigue and was unable to tolerate any physical activity. Her labs again showed elevated inflammatory markers. The patient was prescribed ibuprofen 200 mg three times daily and colchicine 0.6 mg daily. However, these medications did not give any relief. The inflammatory markers continued to rise, and the patient began having intermittent fevers. The patient was advised to go to the ER the following day (day 2) and was diagnosed with recurrent pericarditis. From here on, the day number listed indicates the day since symptom onset of the second incidence of pericarditis. Day 1 indicated the date of symptom onset. She also had a very prominent pericardial rub and painful pleuritic chest pain. Echocardiography in the ER revealed trivial pericardial effusion with normal wall motion. The patient was discharged with instructions to follow up with outpatient cardiology regarding the continued use of colchicine and ibuprofen. 

**Figure 2:**
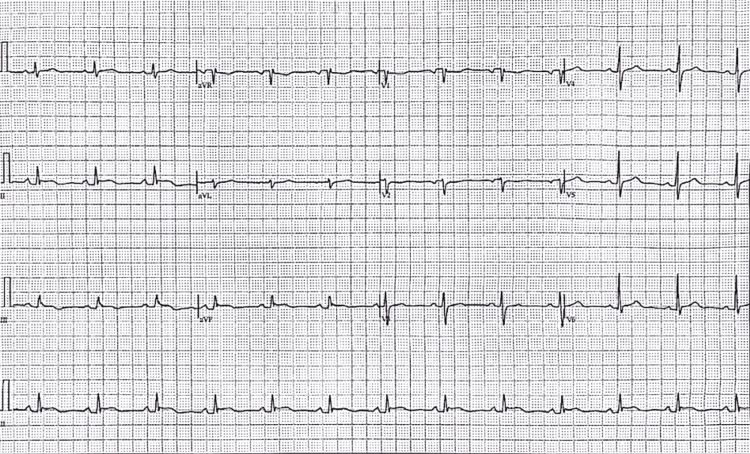
Electrocardiogram at presentation on day 6 showing ST segment elevation followed by a PR depression.

**Figure 3:**
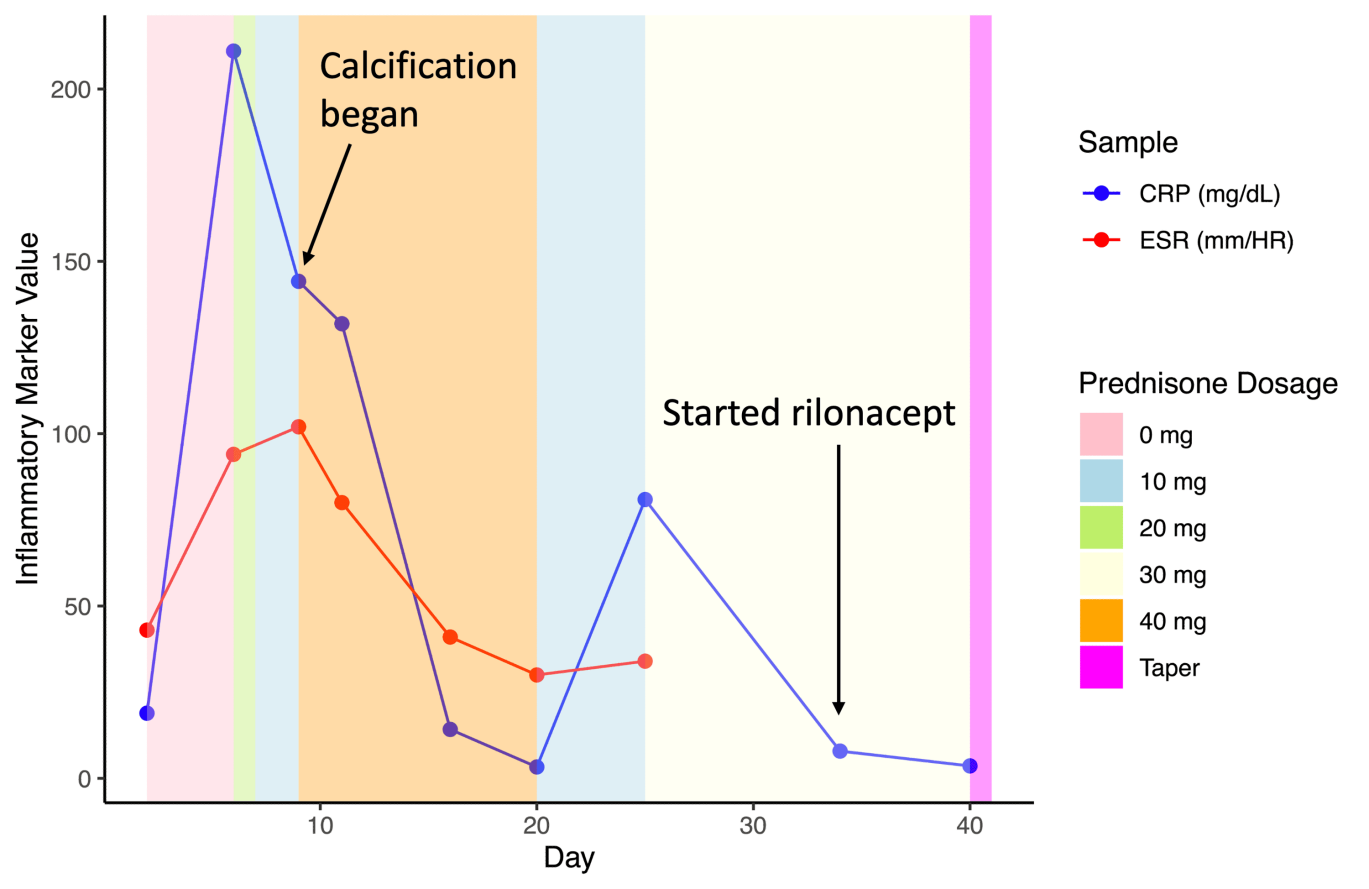
The Inflammatory markers CRP (blue) and ESR (red) fluctuated over time by prednisone dosage (background color) during the second incidence of pericarditis. Day corresponds to the number of days passed since the start of the second incidence of pericarditis. The label *Calcification began* indicates the day when calcification of the pericardium began, and the label *Started rilonacept* indicates when the patient took their first dose of rilonacept. ESR, erythrocyte sedimentation rate; CRP, C-reactive protein

On day 6, EKG revealed an ST segment elevation followed by a PR depression, strongly indicating pericarditis (Figure 2). The patient also had a CT of the abdomen, pelvis, and chest, which showed increasing pericardial effusion and trivial bilateral pleural effusion, which was slightly greater on the left side. Also on day 6, the patient's CRP peaked at 211 mg/dL, and ESR was 94 mm/hour (Figure 3). Due to the extremely elevated CRP and abnormal CT, local doctors at the patient’s rural ER did not feel equipped to manage the patient since they believed her pericarditis was worsening. She was advised to go to a tertiary center ER and was prescribed 20 mg of prednisone to take immediately. Labs done in the ER showed a significant decrease in CRP levels (Figure 3). The patient was prescribed 10 mg of prednisone daily. The patient also had a malignancy and autoimmune workups that were negative. The patient also began calcium and vitamin D supplementation due to the risk of corticosteroids increasing the risk of osteoporosis. 

**Figure 4:**
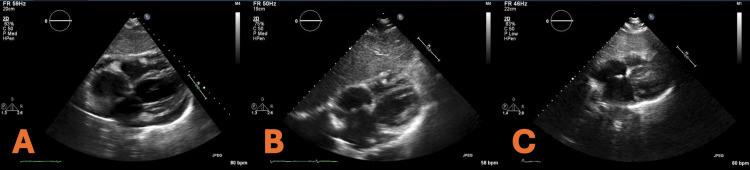
Echocardiograph from day 9 demonstrating early calcification forming on the pericardium and trivial pericardial effusion (A), echocardiograph from day 25 showing worsening calcification of the pericardium (B), and echocardiograph two months after onset of recurrent pericarditis showing resolution of the calcification of the pericardium (C).

**Figure 5:**
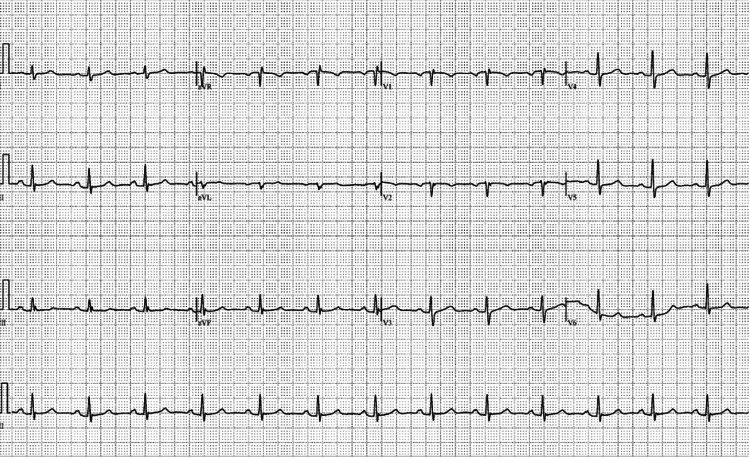
Electrocardiogram (EKG) on day 23 of presentation showing normal results.

On day 9, echocardiography showed early calcification forming on the pericardium (Figure 4A). Inflammatory markers remained high, N-Terminal Pro-B-Type Natriuretic Peptide (NT-proBNP) was 486 pg/mL, ferritin was 337 ng/mL, and liver enzymes were elevated. NT-proBNP is a biomarker of ventricular wall stress and cardiac dysfunction that, while often associated with heart failure, can also be elevated in pericardial inflammation due to increased myocardial strain and, in this case, likely reflects the inflammatory burden on the heart rather than primary myocardial dysfunction. Due to the concerning imaging, high inflammatory markers, and worsening symptoms, the patient was prescribed a higher dose of prednisone (40 mg daily). CRP and ESR decreased over the next few days (Figure 3). On day 19, the patient had a cardiac magnetic resonance imaging (MRI), which showed that both the pericardial and pleural effusions had resolved, and on the following day (day 20), the patient was told to decrease prednisone to 10 mg daily, as all chest pain had subsided and the patient could continue to do daily tasks without cardiac symptoms. On day 23, the patient had a normal EKG, confirming the pericarditis was resolving (Figure 5).

On day 24, the patient began experiencing another round of extreme chest pain despite still being on prednisone. A repeat echocardiography demonstrated worsening pericardial calcification stemming from the intensified pericarditis (Figure 4B). The patient’s CRP rose, which led the patient’s prednisone to be increased to 30 mg daily (Figure 3). Due to the severity of the symptoms and being in a rural setting, the patient was referred by her local cardiologist to a cardiologist in a local urban area who had more experience treating recurrent pericarditis. One month after the onset of recurrent pericarditis, in consultation with the new cardiologist, a biologic medication, rilonacept, was started. She had a loading dose of 320 mg/week and continued with 160 mg/week. Inflammatory markers quickly subsided once the patient began the medication (Figure 3). Due to concerns about the long-term effects of chronic steroid use, such as osteoporosis, a Dual-Energy X-ray Absorptiometry (DEXA) scan was performed, revealing osteopenia. Once a baseline of normalcy was reached with CRP, the patient began a slow eight-week taper of prednisone with close clinical monitoring to assess for signs of recurrence. A repeat echocardiogram performed two months after the onset of recurrence demonstrated a reduction in calcification of the pericardium (Figure 4C). The patient had been on rilonacept for one year and had no signs or symptoms of pericarditis. She continues vitamin D supplementation, and the patient follows up with a rheumatologist every six months to ensure sustained remission and early detection of any potential relapse.

## Discussion

A pericarditis diagnosis can be made by having at least two of the four following criteria: chest pain, pericardial rub, EKG changes (ST-segment elevation and PR-segment depression), and new or worsening pericardial effusion [8]. Epidemiological data on acute pericarditis is not abundant, but few studies point to the diagnosis not being uncommon. One study found that 27.7 patients in 100,000 people were diagnosed with the disease in northern Italy [17]. The prevalence of pericarditis in the United States was estimated to be 40 patients per 100,000 persons, according to data from 2007 to 2016 [18]. In 2024, the American Academy of Family Physicians reported that acute pericarditis was diagnosed in approximately 4.4% of patients presenting to the emergency department for nonischemic chest pain [19]. If pericarditis resolves for a four- to six-week interval and reoccurs, it is known as recurrent pericarditis. Acute pericarditis has a high recurrence rate, with 20% to 30% of people experiencing a consequent episode when colchicine is not utilized [20]. While not all recurrent cases develop resistance, repeated failure of NSAIDs, colchicine, and corticosteroids to resolve symptoms can lead to corticosteroid dependence and the need for alternative treatment options. When traditional therapies fail, biologics, notably IL-1 blockers such as anakinra or rilonacept, are considered, particularly in corticosteroid-dependent cases [1,8]. IL-1 plays a central role in the pathophysiology of pericarditis by promoting inflammation and immune system activation, which contributes to symptom persistence and recurrence. 

Rilonacept is an anti-IL-1 drug that binds both IL-1α and IL-1β [21]. In the literature, there are limited studies on rilonacept as a therapeutic agent for corticosteroid-dependent recurrent pericarditis [14,15]. Specifically, Affas et al. conducted a meta-analysis that identified only two randomized control clinical trial studies investigating rilonacept’s effectiveness in this condition. The majority of existing studies covering rilonacept’s use in pericarditis are small-scale, with a limited number of participants, making it difficult to draw broad conclusions. Despite this, the effect size of these studies was still large and significant [11,13]. These initial findings suggest promising results in reducing recurrence rates and dependency on corticosteroids, highlighting the need for further large-scale research.

The financial burden of rilonacept remains a major concern, as the cost can reach approximately $30,000 per month out of pocket for patients without coverage [22]. While this direct figure may vary by region, insurance status, and assistance programs, access to medication poses a challenge. Cost-effectiveness studies are needed to determine the long-term benefits versus the financial burden, and increased awareness of insurance reimbursement options or manufacturer assistance programs could improve accessibility. Due to the rarity of corticosteroid-dependent acute recurrent pericarditis, prescribing rilonacept with limited clinical data has been uncommon. We hope this report can be used as guidance for future physicians to use in their practices and promote the need for more randomized control trials and clinical trials on rilonacept. Many questions remain on the efficacy of this drug in various stages of pericardial inflammation and how it affects people of varying medical histories and ethnic groups. As such, it is of the utmost importance that more data and reports be published on the use of rilonacept in treating acute, recurrent pericarditis.

In our case, the patient was successfully treated for acute pericarditis using first-line therapeutics at the initial onset but experienced a recurrence eight months later. During this recurrence, symptoms and inflammation were unresponsive to NSAIDs and colchicine, necessitating corticosteroid use. However, tapering off corticosteroids led to rebound inflammation and chest pain, indicating dependence and the need for an alternative treatment (Figure 3). Additionally, the patient developed early pericardial calcification and experienced elevated CRP levels as high as 211 mg/dL. Chronic administration of corticosteroids was relatively contraindicated due to its adverse effects, particularly in this postmenopausal diabetic patient with osteopenia, as prolonged use could exacerbate osteoporosis, blood sugar dysregulation, and dyslipidemia. Clinically significant bone loss has been shown to occur in the majority of patients exposed to corticosteroids, and data show that 30%-50% of patients taking long-term corticosteroids can experience fractures [23]. Corticosteroid-induced alterations in metabolism can also induce hyperglycemia, especially in diabetic patients, which add to the increased risk of cardiovascular disease already seen with diabetes mellitus [24]. 

Rilonacept allowed for successful corticosteroid tapering without rebound inflammation and prevented further pericardial calcification, reducing the risk of constrictive pericarditis. The biological mechanism behind this benefit lies in IL-1 inhibition, which directly reduces inflammatory cytokine activation, thereby mitigating chronic pericardial damage. Rilonacept was chosen over anakinra due to its less frequent dosing schedule, which improves patient adherence, and its longer half-life, allowing for more sustained IL-1 blockade [12,25]. Additionally, its FDA approval for recurrent pericarditis provided greater clinical justification for its use in this case. Additionally, in contrast to corticosteroids, there is no evidence that rilonacept negatively impacts bone density or glucose metabolism, making it a safer long-term option for this patient’s comorbidities. It should be noted that the overlap in the use of rilonacept and corticosteroids complicates determining whether inflammation remission was due to one or both medications. Further research is needed to explore potential interactions between rilonacept and glucocorticoids.

The main side effects of rilonacept reported in the literature include injection site reactions, an increased incidence of upper respiratory infections, and hyperlipidemia [26]. Hyperlipidemia is of particular importance in diabetic patients as it can further increase the risk of cardiovascular disease, so monitoring is recommended just as with chronic corticosteroid use. Special considerations must be made for individuals with diabetes or osteopenia, as infections could pose a greater risk due to immune system modulation. Regular monitoring during treatment, including inflammatory markers, lipid panels, bone density assessments, and infection surveillance, is recommended to ensure patient safety and therapeutic efficacy. Required immunizations should be administered before the initiation of rilonacept therapy, and live vaccines should not be administered while taking the drug [26].

## Conclusions

Acute, recurrent pericarditis is a painful condition that can severely decrease the quality of life and lead to complications such as cardiac tamponade or constrictive pericarditis. In this case, rilonacept was associated with significant clinical improvement, including the resolution of pericardial inflammation and prevention of disease recurrence. However, it is important to acknowledge that this observation does not definitively establish proof of rilonacept’s role in preventing constrictive pericarditis, particularly given concurrent prednisone usage when rilonacept was started. While existing studies and reports suggest the drug's potential in mitigating disease progression, further research is necessary to determine its efficacy across diverse populations and varying severities of pericarditis. This case highlights the need for more robust data to clarify rilonacept’s role in pericarditis management, especially in corticosteroid-dependent patients with early pericardial calcification. Continued investigation will be essential in identifying safe and effective treatment strategies for all affected patients.
